# Lapatinib access into normal brain and brain metastases in patients with Her-2 overexpressing breast cancer

**DOI:** 10.1186/s13550-015-0103-5

**Published:** 2015-04-30

**Authors:** Azeem Saleem, Graham E Searle, Laura M Kenny, Mickael Huiban, Kasia Kozlowski, Adam D Waldman, Laura Woodley, Carlo Palmieri, Charles Lowdell, Tomomi Kaneko, Philip S Murphy, Mike R Lau, Eric O Aboagye, Raoul C Coombes

**Affiliations:** 1 Imanova Centre for Imaging Sciences, Imperial College London, Hammersmith Hospital, Burlington Danes Building, Du Cane Road, London, W12 0NN UK; 2 Department of Surgery and Cancer, Imperial College London, Charing Cross Hospital, Fulham Palace Road, London, W6 8RF UK; 3 Division of Brain Sciences, Imperial College Department of Imaging, Imperial College Healthcare NHS Trust, Charing Cross Hospital, Fulham Palace Road, London, W6 8RF UK; 4 Department of Surgery and Cancer, Imperial College London, Hammersmith Hospital, Du Cane Road, London, W12 0NN UK; 5 Department of Molecular and Clinical Cancer Medicine, Duncan Building, Daulby Street, Liverpool, L69 3GA UK; 6 Imperial College Healthcare NHS Trust, Charing Cross Hospital, Fulham Palace Road, W6 8RF London, UK; 7 GlaxoSmithKline Oncology, Stockley Park West, Uxbridge, Middlesex, UB11 1BT UK; 8 Clinical Imaging and Medicines Development, GlaxoSmithKline, Gunnels Wood Road, Stevenage, Hertfordshire SG1 2NY UK

**Keywords:** Lapatinib bio-distribution in brain metastases, Her-2-positive breast cancer, PET imaging, Blood-brain barrier

## Abstract

**Background:**

Brain metastases are common in human epidermal growth factor receptor (Her)-2-positive breast cancer. Drug access to brain metastases and normal brain is key to management of cranial disease. In this study, positron emission tomography (PET) scanning after administration of radiolabelled lapatinib was used to obtain direct evidence of cranial drug access.

**Methods:**

Patients with Her-2+ metastatic breast cancer either with at least one 1-cm diameter brain metastasis or without brain metastases underwent dynamic carbon-11 radiolabelled lapatinib ([^11^C]lapatinib)-PET. Less than 20 μg of [^11^C]lapatinib was administered before and after 8 days of oral lapatinib (1,500 mg once daily). Radial arterial blood sampling was performed throughout the 90-min scan. The contribution of blood volume activity to the tissue signal was excluded to calculate lapatinib uptake in normal brain and metastases. Partitioning of radioactivity between plasma and tissue (*V*_T_) was calculated and the tissue concentration of lapatinib derived. Plasma lapatinib levels were measured and adverse events noted.

**Results:**

Six patients (three with brain metastases) were recruited. About 80% plasma radioactivity corresponded to intact [^11^C]lapatinib after 60 min. PET signal in the brain corresponded to circulating radioactivity levels, with no [^11^C]lapatinib uptake observed in normal brain tissue. In contrast, radioactivity uptake in cranial metastases was significantly higher (*p* = 0.002) than that could be accounted by circulating radioactivity levels, consistent with [^11^C]lapatinib uptake in brain metastases. There was no difference in lapatinib uptake between the baseline and day 8 scans, suggesting no effect of increased drug access by inhibition of the drug efflux proteins by therapeutic doses of lapatinib.

**Conclusions:**

Increased lapatinib uptake was observed in brain metastases but not in normal brain.

**Trial registration:**

ClinicalTrials.gov: NCT01290354

## Background

Overexpression of human epidermal growth factor receptor (Her)-2 in breast cancer is considered an independent factor for development of brain metastases [1] with up to 37% of patients with Her-2-positive disease relapsing intracranially despite control of extra-cranial metastatic disease [2]. Possible reasons for the increased incidence of brain metastases include the aggressive nature of Her-2-positive disease, increased ability of Her-2 cells to survive and/or home to the brain and inability of drugs to pass an intact blood-brain barrier (BBB) [2,3]. It has been hypothesised that in contrast to the large monoclonal antibodies, the small molecule lapatinib (Tykerb/Tyverb; GlaxoSmithKline, Brentford, UK; molecular weight: 581.07), an oral dual epidermal growth factor receptor and Her-2 inhibitor, may cross the BBB. In addition, since lapatinib is a P-glycoprotein (Pgp) and breast cancer resistance protein (BCRP) substrate, therapeutic doses of lapatinib may inhibit drug efflux by blocking Pgp/BCRP [4], thereby enhancing its access into the brain through an intact BBB.

**Figure 1:**
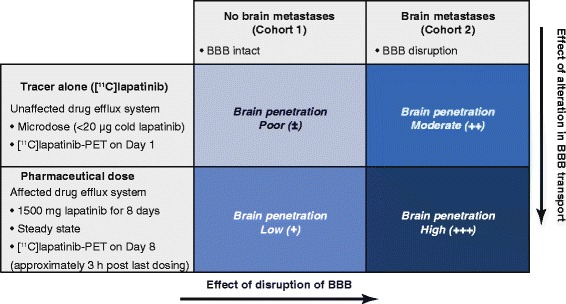
Study hypothesis. It was hypothesised that brain penetration in normal brain would increase with therapeutic serum concentrations of lapatinib due to the effect of lapatinib on drug efflux pumps. Higher brain penetration in metastases compared with normal brain was also hypothesised due to the disruption of the BBB in cranial metastases. ^11^C, carbon-11 radiolabelled; BBB, blood-brain barrier; PET, positron emission tomography.

In order to evaluate lapatinib access into normal brain and brain metastases, a positron emission tomography (PET) study was performed with carbon-11 radiolabelled lapatinib ([^11^C]lapatinib) in patients with Her-2-positive breast cancer. Further, in order to test our hypothesis that therapeutic doses of lapatinib increase brain access into the brain and brain metastases, by blockage of the drug efflux pump, paired [^11^C]lapatinib-PET imaging in patients before and after therapeutic doses of lapatinib was performed. A schematic representation of our study hypothesis is shown in Figure 1.

## Methods

### Study design

An open-label study was performed in patients with Her-2-positive breast cancer with no brain metastases (cohort 1) and with at least a single brain metastases ≥1 cm (cohort 2) as confirmed by MRI. Other inclusion criteria included female patients aged at least 18 years with histologically or cytologically confirmed advanced or metastatic breast cancer with overexpression of Her-2, Eastern Cooperative Oncology Group (ECOG) performance status of 0–2, stable condition as judged by the investigator and adequate hepatic and renal function. An Allen’s test to check the adequacy of collateral circulation of the hand was also performed at screening as radial arterial cannulation was performed on the day of the scan. The protocol stipulated that patients were not allowed to receive concurrent treatment with an investigational medicinal product or anti-cytotoxic therapy whilst on the imaging part of the study apart from the use of anastrozole. Patients were required a washout period of 4 weeks and 30 days after previous radiotherapy or chemotherapy, respectively. Up to 1.5 mg of dexamethasone was allowed whilst patients were on the imaging part of the study. Two PET-computed tomography (CT) scans were performed after administration of a microdose (<20 μg) of [^11^C]lapatinib, (i) at baseline (day 1) in lapatinib-naïve patients and (ii) after 7 days of 1,500 mg of oral lapatinib daily, starting on day 1 after completion of the day 1 PET scan. The day 8 PET scan started at 3 h post-dosing of oral lapatinib to coincide with the known maximal plasma lapatinib level (*C*_max_). Approval was obtained from the West London Research Ethics Committee (10/H0707/93) to conduct this PET imaging study in this patient group with metastatic disease during the ‘window’ prior to starting lapatinib-based therapy. Approval was also obtained from the UK Administration of Radioactive Substances Advisory Committee. The study was registered with the European Union Clinical Trials database (EudraCT 2009-009884-76), National Institute of Health database (NCT01290354) and the National Cancer Research Network study portfolio (NCRN262).

### Patients

Demographics and disease characteristics of the eight patients recruited to the study are detailed in Table 1. Six patients (three with and three without brain metastases) underwent both baseline and day 8 [^11^C]lapatinib-PET scans, except one who was unable to have the baseline scan due to radiochemistry failure (tumour receptor status and injected radioactivity and mass for patients scanned are summarised in Tables 2 and 3, respectively). In another subject, the baseline scan was not assessable due to tissue extravasation of the radiotracer. All three patients with brain metastases had completed prior cranial radiotherapy about 5, 11 and 70 weeks prior to the baseline PET scan (Table 3). Optional extra-cranial static PET scans were obtained for six PET sessions, approximately 95 to 100 min after [^11^C]lapatinib injection to evaluate the uptake of [^11^C]lapatinib in sites of extra-cranial metastases and normal tissue within the field of view of the optional scan. Two of the eight patients recruited were unable to undergo [^11^C]lapatinib-PET scans due to inability to reschedule scans after radiochemistry failure and due to regulatory issues. As it was unethical to delay start of their therapy to accommodate the [^11^C]lapatinib-PET scans, both the subjects started their lapatinib therapy on time, as planned.

**Table 1:** **Patient demographics and disease characteristics of all patients recruited**

**Demographic characteristics**	**Value**
Age in years, mean (range)	55.9 (42 to 79)
Race, *n* (%)	
White	4 (50)
African heritage	2 (25)
Asian - Japanese/East Asian/Southeast Asian heritage	1 (13)
Asian - Central/South Asian heritage	1 (13)
Baseline ECOG performance status, *n* (%)	
0	6 (77)
2	2 (25)
Time since diagnosis (years), median (range)	4.5 (1 to 9)

Of the eight patients recruited, only six patients underwent PET. ECOG, Eastern Cooperative Oncology Group; PET, positron emission tomography.

**Table 2:** **Tumour receptor status of patients imaged in the study**

**Subject number**	**HER-2 status (IHC)**	**Oestrogen receptor (ER) status**	**Progesterone receptor (PR) status**
1	3+	ER negative	PR negative
3	3+	ER negative	PR positive
4	3+	ER positive	PR negative
7	2+	ER positive	PR negative
9	3+	ER positive	PR positive
10	3+	ER negative	PR negative

IHC, immunohistochemistry.

**Table 3:** **Patient injected radioactivity and mass of [** ^**11**^ **C]lapatinib**

**Subject number**	**Brain metastases**	**Time lapse after previous cranial RT (weeks)**	**Scan day**	**Injected activity (MBq)**	**Radiotracer mass (μg)**
1	No	NA	Baseline	44	1.16
			Day 8	148	4.45
3	No	NA	Baseline	69	4.47
			Day 8	288	5.21
4	Yes	70	Baseline	104	2.26
			Day 8	229	3.77
7	Yes	5	Baseline	349	12.39
			Day 8	167	6.85
9	No	NA	Baseline	222^a^	6.73
			Day 8	139	5.50
10	Yes	11	Baseline	-^b^	-
			Day 8	114	4.65

^a^Data not included in analysis due to tissue extravasation of radiotracer injection. ^b^Subject was unable to have baseline scan due to radiochemistry failure. MBq, megabecquerel; NA, not applicable as patients did not have brain metastases.

### Radiochemistry

**Figure 2:**
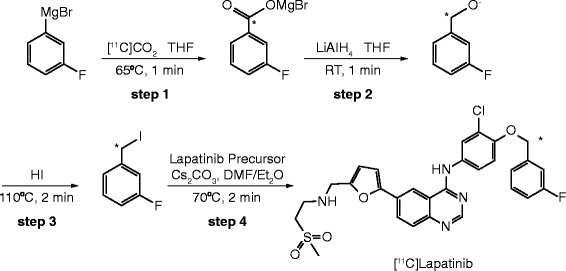
Fully automated and good manufacturing process-compatible synthesis developed to label lapatinib with radiolabelled carbon-11 in the benzylic position. [^11^C]Lapatinib was prepared in a two-pot four-step synthesis, with intermediate preparation of [^11^C]-3-fluorobenzyl iodide that was reacted in the last step with the lapatinib precursor. ^11^C, carbon-11.

[^11^C]Lapatinib was prepared in a two-pot four-step synthesis, with intermediate preparation of [^11^C]-3-fluorobenzyl iodide (Figure 2). Cyclotron-derived [^11^C]CO_2_ was first reacted with 3-fluorophenylmagnesium bromide in tetrahydrofuran. The resulting acid was reduced with lithium aluminium hydride, followed by iodination reaction in the presence of hydriodic acid. The obtained [^11^C]-3-fluorobenzyl iodide was intermediately purified by solid-phase extraction, before being reacted with the lapatinib precursor in dimethylformamide in the presence of caesium carbonate. Purification of [^11^C]lapatinib was achieved by reverse-phase high-performance liquid chromatography (HPLC). The fraction containing the product was formulated into 20% ethanolic saline by solid-phase extraction, followed by filtration through a 0.2-μm Pall Tuffryn® membrane (Pall Corporation, Port Washington, NY, USA). Quality control methods for clinical batches of [^11^C]lapatinib were developed in accordance with the European Pharmacopoeia guidelines.

### PET scanning procedure and blood sampling

Following a low-dose CT scan for attenuation correction, dynamic PET scans lasting 90 min were performed on a Siemens HiRez 6 PET-CT scanner (Siemens, Munich, Germany) after intravenous administration of [^11^C]lapatinib. PET data were reconstructed using filtered back projection with corrections for attenuation, scatter, randoms and dead time, into 26 frames of the following duration: 8 × 15 s, 3 × 60 s, 5 × 120 s, 5 × 300 s and 5 × 600 s. Continuous (5 mL/min for 15 min) and discrete (from 5 to 90 min after injection) sampling of radial arterial blood was performed throughout the PET scan for radioactivity and radioactive metabolite analyses. Whole-blood and plasma radioactivity measurements were performed on a PerkinElmer 1470 Wizard well counter (PerkinElmer, Waltham, MA, USA). An Agilent 1200 HPLC analytical system (Agilent Technologies, Inc., Santa Clara, CA, USA) was used to determine the fraction of radioactivity corresponding to the intact parent compound. Additional hourly blood samples from 1 to 6 h after oral lapatinib dosing were assessed on the day 8 scan. Radiation dosage to patients in the study from exposure to the radiopharmaceutical and the attenuation CT scan of the head was 6.5 mSv, equivalent to approximately 3 years of background radiation exposure in the UK. An optional extra-cranial static PET for 10 min was performed in some of the patients.

### Patient monitoring

Fourteen days after the second PET scan, patients were reverted to standard hospital treatment by their oncologist which in most instances was lapatinib and capecitabine. Adverse events (AEs) were noted starting from the baseline scan to 14 days after the second scan; after this, AEs were not actively sought but noted on patient self-reporting.

### Data analysis

Tumour and normal brain regions of interest manually drawn using Analyze® software were applied to dynamic PET images to obtain time versus radioactivity curves (TACs), which were corrected for radioactive decay and normalised for injected radioactivity and patient’s body weight. Tissue uptake (standardised uptake value) and exposure (area under the TAC (AUC)) were calculated. Novel methods were developed to exclude the effects of blood volume signal from images. As the blood volume fraction in metastases cannot be assumed, kinetic modelling was performed to fit the blood volume fraction. This modelling was performed on a voxel-by-voxel basis to remove any assumptions about region boundaries and does not assume homogeneity of blood volume. The fraction of PET signal enabled dissection of the total signal into blood and non-blood components and permitted visual evaluation of metastases in the PET image. Kinetic tissue modelling was performed using in-house MIAKAT^™^ software implemented using Matlab R2008b (The MathWorks Inc., Natick, MA, USA) to obtain PET volume of distribution (*V*_T_) [5]. Statistical *t*-tests were performed to evaluate differences in tissue exposure.

## Results

### PET scanning

The scanning procedure was well tolerated by all patients. Apart from some soreness and self-resolving mild local swelling, there were no local consequences in the patient who experienced extravasation of the radiotracer. There were no other acute side effects related to the administration of [^11^C]lapatinib. The most common AEs observed after completion of the imaging part of the study were diarrhoea (88%), vomiting (50%) and nausea (38%), in keeping with the AE profile of oral lapatinib. The overall recruitment of patients to the study lasted 17 months.

### Radiochemistry productions

There were 4/15 (27%) radiochemistry production run failures, mostly equipment-related, due to the complexity of the fully automated process. The mean (standard deviation (SD)) radiochemical yield generated for quality control was 1,286 (572) MBq, with the high variability due to sensitivity of most of the reagents used and the complexity of the synthesis. Although the radioactivity released for injection was 492 (201) MBq, the sticky nature of the [^11^C]lapatinib solution leading to its adherence to the injection syringe and line resulted in lower administered activity (170 (93) MBq; SD (Table 3)). The mean radiochemical purity was 100%, and the mean (SD) mass and specific activity of [^11^C]lapatinib was 5.22 (2.92) μg and 66 (24) GBq/μmol, respectively.

### Blood data

#### Radioactivity data

Arterial blood data obtained from all scans revealed a mean radioactivity in plasma of 83% at 60 min which corresponded to [^11^C]lapatinib with minimal variability allowing generation of population [^11^C]lapatinib fraction. Individually measured blood and plasma data were used with the population parent fraction curve to generate the required plasma parent input function.

#### Plasma lapatinib on day 8

Pharmacokinetic parameters were variable, spanning a fourfold range of values, consistent with previous published data [6]. The maximum observed concentrations (*C*_max_) were 911 to 4,121 (mean 2,592) ng/mL and varied 1.4 to 14.7-fold over the pre-dose concentration (range 136 to 4,121 ng/mL; mean 445 ng/mL). Lag times in absorption ranged from 0 to 4 h post-dose. The absorption phase appeared to be complete by 6 h (*T*_max_) in all but two patients (patients 3 and 7); in these two patients, actual *C*_max_ may be higher and *T*_max_ later than the observed values.

### Imaging data

#### Semi-quantitative tissue uptake

**Figure 3:**
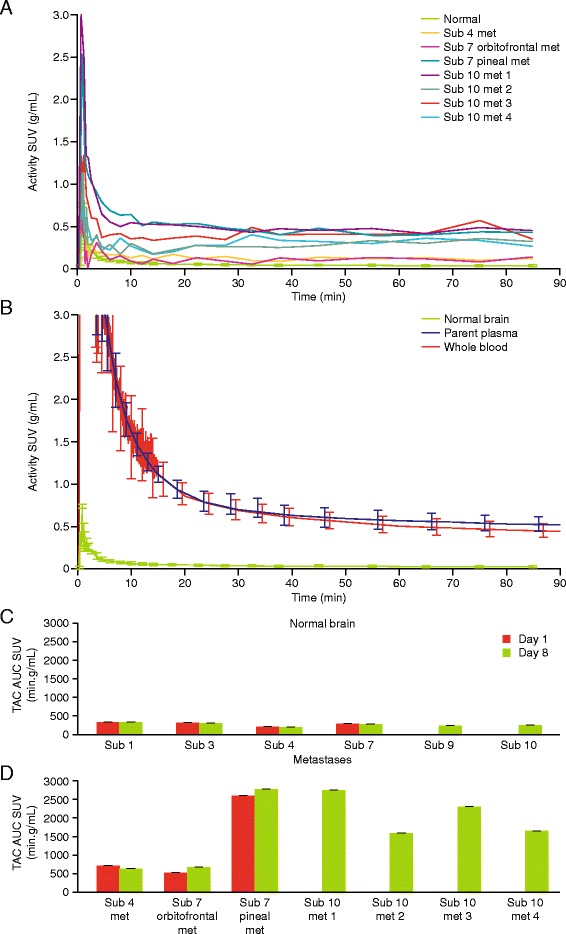
Radioactivity versus time curves and tissue exposure. Time-activity curves (TACs) for individual metastases for all the subjects from the day 8 PET scan **(A)** show variability in uptake between and within patients. Mean TAC is also shown for comparison (green) and shows minimal variability. In **(B)**, mean TACs for normal brain (green) is plotted for comparison with mean whole blood (red) and plasma (plasma) TACs. Uptake has been corrected for injected activity and normalised for body weight and quantified in the *Y*-axis as standardised uptake value (SUV; g/mL). Tissue exposure (area under the TAC SUV (min.g/mL) for normal brain **(C)** and metastases **(D)** shows variability in uptake in metastases. However, there is minimal variability in uptake within metastases on day 1 (red bars) compared to day 8 (green bars). Minimal variability in lapatinib uptake is seen in normal brain between subjects. AUC, area under the curve concentration; contrib, contribution; min, minutes; Sub, subject; SUV, standardised uptake value; TAC, time-averaged concentration.

Mean TAC for normal brain from the day 8 scans plotted with individual TACs for brain metastases (Figure 3A) and with mean whole blood and plasma [^11^C]lapatinib TAC (Figure 3B) illustrates the high variability of uptake in metastases compared with normal brain. No difference in within-tissue exposure (AUC) was observed between days 1 and 8 in normal brain and metastases. However, tissue exposure was significantly higher in metastases compared with normal brain (*p* = 0.002 (Figure 3C,D and Table 4)).

**Table 4:** **Tissue exposure (AUC) for the duration of the PET scan**

**Normal brain**	**AUC (min.g/mL)**	**Tumour metastases**	**AUC (min.g/mL)**
Sub 1 - BL	319.6318	Sub 4 met - BL	708.198
Sub 1 - D 8	328.7575	Sub 4 met - D 8	623.23
Sub 3 - BL	304.0247	Sub 7 met 1 - BL	518.227
Sub 3 - D 8	301.1074	Sub 7 met 1 - D 8	665.909
Sub 4 - BL	203.6962	Sub 7 met 2 - BL	2,584.31
Sub 4 - D 8	198.8753	Sub 7 met 2 - D 8	2,757.86
Sub 7 - BL	289.1109	Sub 10 met 1 - D 8	2,731.19
Sub 7 - D 8	272.9378	Sub 10 met 2 - D 8	1,577.81
Sub 9 - D 8	233.2629	Sub 10 met 3 - D 8	2,289.3
Sub 10 - D 8	252.7335	Sub 10 met 4 - D 8	1,647.02

AUC, area under the curve concentration; BL, baseline; D, day; met, metastases; min, minutes; PET, positron emission tomography; Sub, subject.

#### Quantification of uptake with model-independent methods

**Figure 4:**
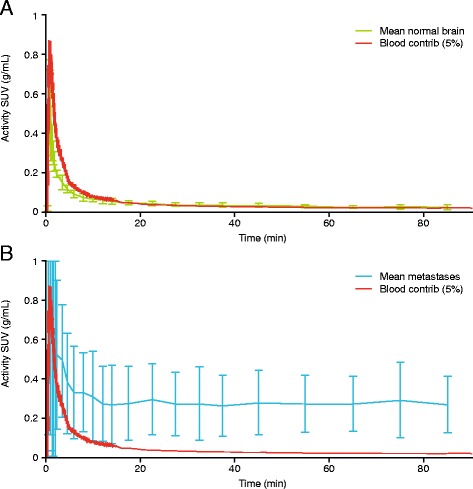
Blood volume contribution to activity to intracerebral uptake. Mean radioactivity versus time curves for **(A)** normal brain and **(B)** brain metastases and scaled-down blood radioactivity versus time curves to illustrate cerebral blood volumes of 5%. Sub, subject; SUV, standardised uptake value; min, minutes.

To verify if [^11^C]lapatinib crossed an intact BBB, the contribution of blood volume to the PET was corrected by scaling the tissue TACs assuming a 5% cerebral blood volume [7] and compared with arterial TACs. In contrast to normal brain TACs which were identical to blood TACs (Figure 4A), uptake in metastases was higher than that accounted by blood volume only (Figure 4B), confirming that the PET signal in normal brain was due to the blood volume and not due to access of [^11^C]lapatinib in to the brain tissue.

**Figure 5:**
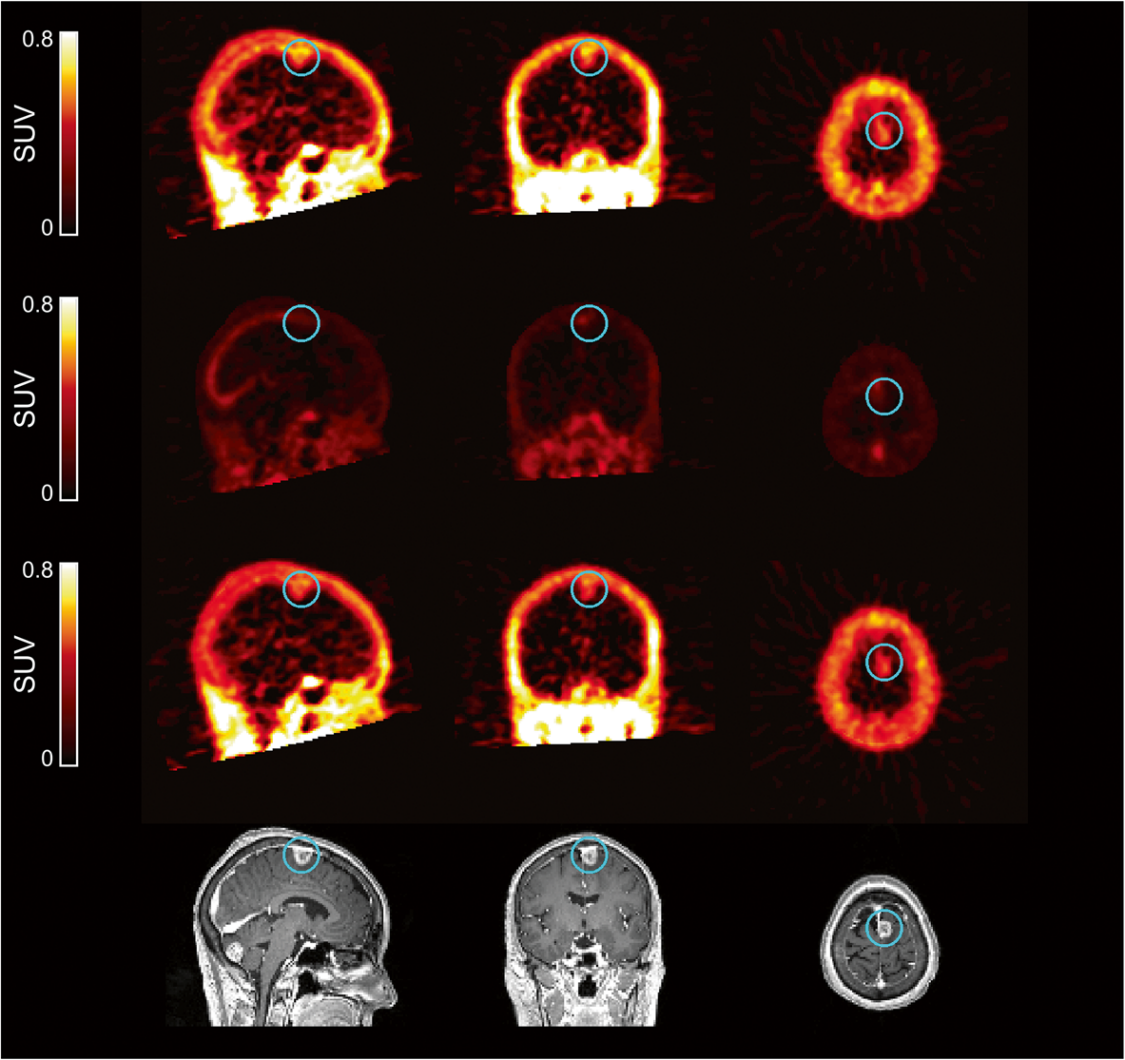
Image data for patient 10. The image data show radioactivity distribution in normal brain and cerebral metastases (enclosed in blue circle) (top panel) and are separated into non-blood (middle upper panel), blood (middle lower panel) and corresponding contrast-enhanced MRI images (bottom panel). Since the blood volume in metastases may not be 5%, a blood volume fraction model was fitted to dynamic data on a voxel-by-voxel basis. The uptake of radioactivity in the brain metastases was higher than that contributed from a model-fitted blood volume. MRI, magnetic resonance imaging; SUV, standardised uptake value.

However, since the blood volume in metastases may be higher and not 5% as in normal brain, a blood volume fraction model was fitted to dynamic data, which permitted dissection of the PET image data into fitted non-blood and blood components and allowed visual assessment (Figure 5). These images show that the uptake of radioactivity in the brain metastases (highlighted in blue circle) is higher than that contributed from a model-fitted blood volume, suggesting that the observed uptake of [^11^C]lapatinib in brain metastases cannot be attributed to blood volume alone.

#### Kinetic modelling of tissue data

As normal brain tissue signal was adequately described as a simple blood volume component, no further modelling was required. Metastases TACs were noisy and thus described adequately by several models, with the most appropriate being a one-tissue compartment model for reversible binding, with PET *V*_T_ estimates ranging from 0.16 to 1.03 with no significant differences in *V*_T_ or *K*_1_ (the other model parameter) between baseline and day 8 scans. Therefore, assuming equilibrium conditions, lapatinib concentrations in metastases between 300 and 2,000 ng/mL (0.52 to 3.44 μM) were estimated by multiplying the PET *V*_T_ by the plasma lapatinib concentration on day 8.

#### [^11^C]lapatinib uptake in other tissues

[^11^C]Lapatinib uptake was observed in tissues such as muscle and bone (Figure 5) that were within the FoV of the cranial PET scans. However, due to the short half-life of carbon-11 (20 min), the *in vivo* radioactivity was reduced to <4% of the administered activity by the time (approximately 100 min) the optional extra-cranial scans were completed. This, combined with biological washout, meant that the images contained relatively high noise compared with the signal. Uptake was observed in extra-cranial tumours, although the signal quality was insufficient to support quantitative analysis. Higher uptake on visual inspection was primarily observed in the liver and gallbladder when they were in the scanner’s field of view.

## Discussion and conclusions

In this study, we have clearly demonstrated that there is uptake of lapatinib in brain metastases that cannot be attributed to blood volume effects alone. The extent of uptake was highly variable between the metastases, consistent with preclinical rodent studies [8] and clinical data from Morikawa et al*.* which showed a 60-fold variability of lapatinib uptake (1 to 63 μM) in resected brain metastases of four patients after oral lapatinib [9]. Although the sevenfold variability (300 to 2,000 ng/mL; 0.5 to 3.4 μM) in metastatic uptake observed in our study was lower, which may be due to some imprecision in our estimation due to low biological uptake and small volume of the metastases sampled, adequate fitting of kinetic models was obtained. Nevertheless, lapatinib concentrations in brain metastases in our study were comparable to those obtained by Morikawa et al. [10] and within the range of the half maximal inhibitory concentration (IC_50_) for breast cancer cell lines (0.025 to 5 μM) [4]. However, the variable lapatinib uptake in metastases demonstrates the challenge posed by tumour heterogeneity [9], a barrier for optimisation of therapy as demonstrated by the modest single-agent clinical activity [11].

In contrast to Morikawa et al., we were also able to evaluate lapatinib uptake in normal brain and observed a consistent lack of lapatinib uptake in normal brain, in patients with and without brain metastases. The radioactivity observed in normal brain was due to brain vasculature activity and not due to brain tissue uptake. Our hypothesis that therapeutic levels of lapatinib [4] may increase lapatinib brain uptake by acting as a substrate for drug efflux proteins was disproved in normal brain and metastases. This would imply that the utility of lapatinib as prophylactic therapy that aimed to exploit the study hypothesis is likely to be futile. However, from the study design, it is not possible (nor did we aim) to investigate lapatinib access into normal brain in combination with other cytotoxic agents or agents that target the BBB [12,13]. Since the uptake of [^11^C]lapatinib coincides with regions of enhanced signal on gadolinium magnetic resonance relative to areas of no uptake, we have established that lapatinib uptake in brain metastases is mainly a result of local BBB impairment in the metastases. This confirms the preclinical data which similarly demonstrated that uptake in brain metastases correlated with altered BBB permeability and that uptake in normal brain was very minimal even at higher doses of lapatinib suggesting that higher doses of lapatinib were unable to block the drug efflux mechanisms, as anticipated [8,14].

We observed bone uptake that may be consistent with Her-2 expression in haematopoietic cells [15]. Despite the poor image quality of extra-cranial PET, increased uptake in the liver and gallbladder was consistent with the known hepatic metabolism of lapatinib.

Management of cranial disease remains a challenge in patients with Her-2-positive breast cancer and is an unmet need [2]. Although lapatinib and capecitabine in combination demonstrated activity in patients with previously untreated Her-2-positive brain metastases [12], the CEREBEL study was unable to demonstrate the lack of superiority for lapatinib over trastuzumab in the prevention of brain metastases [16]. Therefore, this study if it had been performed earlier would possibly have informed an alternative design of the CEREBEL study. This also highlights the importance of planning and conducting such small but important translational imaging studies early in the rational development of drugs.

Despite the value demonstrated, such studies require considerable radiochemistry development to quality clinical standards, are technically and logistically challenging and are restricted to specialised centres. However, the highly sensitive and quantitative nature of PET provided conclusive answers with six patients to facilitate decision-making in the clinical development of a drug asset. Significantly, if such answers are provided early, this may result in cost-saving and rational drug development.

## Key message

Patients with Her-2-positive breast cancer have higher incidence of brain metastases. PET scans were done after administration of carbon-11 radiolabelled lapatinib to investigate intracranial lapatinib access. Lapatinib uptake was observed in brain metastasis, but not in normal brain, suggesting that lapatinib may have a role in the treatment of brain metastases but not in its prevention.

## Abbreviations

AUC: area under the time-activity curve
BBB: blood-brain barrier
FoV: field of view
IC_50_: half maximal inhibitory concentration
MBq: megabecquerel
PET: positron emission tomography
SD: standard deviation
SUV: standardised uptake value
TACs: time versus radioactivity curves
*V*_T_: PET volume of distribution.
