# Erratum to: Lapatinib access into normal brain and brain metastases in patients with Her-2 overexpressing breast cancer

**DOI:** 10.1186/s13550-017-0323-y

**Published:** 2017-09-08

**Authors:** Azeem Saleem, Graham E. Searle, Laura M. Kenny, Mickael Huiban, Kasia Kozlowski, Adam D. Waldman, Laura Woodley, Carlo Palmieri, Charles Lowdell, Tomomi Kaneko, Philip S. Murphy, Mike R. Lau, Eric O. Aboagye, Raoul C. Coombes

**Affiliations:** 1 Imanova Centre for Imaging Sciences, Imperial College London, Hammersmith Hospital, Burlington Danes Building, Du Cane Road, London, W12 0NN UK; 2 Department of Surgery and Cancer, Imperial College London, Charing Cross Hospital, Fulham Palace Road, London, W6 8RF UK; 3 Division of Brain Sciences, Imperial College Department of Imaging, Imperial College Healthcare NHS Trust, Charing Cross Hospital, Fulham Palace Road, London, W6 8RF UK; 4 Department of Surgery and Cancer, Imperial College London, Hammersmith Hospital, Du Cane Road, London, W12 0NN UK; 5 Department of Molecular and Clinical Cancer Medicine, Duncan Building, Daulby Street, Liverpool, L69 3GA UK; 6 Imperial College Healthcare NHS Trust, Charing Cross Hospital, Fulham Palace Road, London, W6 8RF UK; 7 GlaxoSmithKline Oncology, Stockley Park West, Uxbridge, Middlesex, UB11 1BT UK; 8 Clinical Imaging and Medicines Development, GlaxoSmithKline, Gunnels Wood Road, Stevenage, Hertfordshire, SG1 2NY UK

## Erratum

Since publication of their article [1], the authors have become aware that there is an error in the description of the image data in the legend to Fig. 5. The second sentence of the legend should read as follows:

“The image data show radioactivity distribution in normal brain and cerebral metastases (enclosed in blue circle) (top panel) and are separated into blood (middle upper panel), non-blood (middle lower panel) and corresponding contrast-enhanced MRI images (bottom panel).”
